# A Girl with PRRT2 Mutation Presenting with Benign Familial Infantile Seizures Followed by Autistic Regression

**DOI:** 10.1155/2024/5539799

**Published:** 2024-02-16

**Authors:** Li Zhang, Zhen-Xia Wan, Jin-Yi Zhu, Hui-Juan Liu, Jin Sun, Xiao-Hui Zou, Ting Zhang, Yan Li

**Affiliations:** ^1^Research Center for Child Health, Department of Child Health Care, Key Laboratory of Birth Regulation and Control Technology of National Health Commission of China, Shandong Provincial Maternal and Child Health Care Hospital Affiliated to Qingdao University, Jinan, China; ^2^Neonatal Intensive Care Unit, Key Laboratory of Birth Regulation and Control Technology of National Health Commission of China, Shandong Provincial Maternal and Child Health Care Hospital Affiliated to Qingdao University, Jinan, China; ^3^Affiliated Hospital of Weifang Medical University, School of Clinical Medicine, Weifang Medical University, Weifang, China; ^4^Department of Child Health Care, The First Affiliated Hospital of Shandong First Medical University & Shandong Provincial Qianfoshan Hospital, Jinan, China

## Abstract

Benign familial infantile seizure (BFIS) is an autosomal dominant infantile-onset epilepsy syndrome with a typically benign prognosis. It is commonly associated with heterozygous mutations of the *PRRT2* gene located on chromosome 16p11.2. The frameshift heterozygous mutation (c.649dupC, p.Arg217Profs^*∗*^8) in *PRRT2* is responsible for the majority of BFIS cases. In this report, we present a rare case of a girl with a confirmed clinical and genetic diagnosis of BFIS due to a frameshift heterozygous mutation in *PRRT2* (c.649dupC). She exhibited typical neurodevelopment until 15 months of age, followed by an unexpected severe autistic regression. In addition to BFIS, *PRRT2* mutations are also associated with paroxysmal kinesigenic dyskinesia (PKD) and infantile convulsions and paroxysmal choreoathetosis (ICCA), indicating a complex genotype-phenotype heterogeneity in *PRRT2* mutations. This clinical observation highlights the possibility that BFIS patients with *PRRT2* mutations may not always have a benign neurodevelopmental prognosis, emphasizing the need for long-term clinical follow-up.

## 1. Introduction

Benign familial infantile seizure (BFIS) is an autosomal-dominant epileptic syndrome characterized by infantile-onset, spontaneous remission by two years of age, a positive response to conventional antiepileptic drugs, normal psychomotor developmental outcome, and occasional co-occurrence with infantile convulsions and paroxysmal choreoathetosis (ICCA) and paroxysmal kinesigenic dyskinesia (PKD) [1]. While there is genetic heterogeneity in BFIS, a frameshift heterozygous mutation in *PRRT2* (c.649dupC, p.Arg217Profs^*∗*^8) on chromosome 16p11.2 has been identified in most BFIS cases, with or without PKD or ICCA presentations [1, 2]. However, given the typical benign neurodevelopmental outcomes in BFIS, occurrences of BFIS with recurrent *PRRT2* frameshift heterozygous mutations leading to autism spectrum disorder (ASD) are rare.

Here, we report a case of a girl diagnosed with BFIS based on clinical symptoms and a family history of convulsions, as well as genetically with a frameshift heterozygous mutation (c.649dupC, p.Arg217Profs^*∗*^8), in the *PRRT2* gene. Initially, she displayed symptoms of BFIS and reached typical developmental milestones before 15 month of age but subsequently experienced an unexpected progression to cognitive and autistic regression.

## 2. Narrative

The proband was a 2.5-year-old girl with normal birth and neonatal history. She was the second child of nonconsanguineous healthy parents. At 5 months of age, she experienced her first afebrile seizure following a bout of diarrhea. The seizure was characterized by staring, eye deviation to the left, cyanosis, dribbling foam-like saliva, and bilateral jerks. This episode lasted about one minute and was followed by post-ictal sleep. Subsequently, she experienced another afebrile seizure 3-4 hours after awakening. The initial seizure episode recurred 3-4 times per day over a period of 3-4 days. Following this, she continued to have recurrent afebrile seizures, often in association with colds or diarrhea, with a similar frequency and duration as the initial seizure.

**Figure 1:**
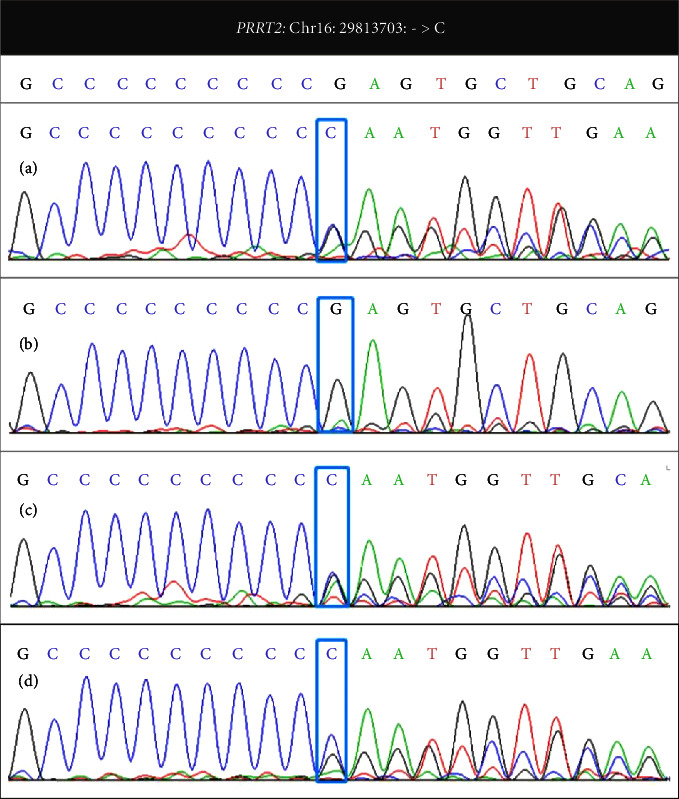
Whole-exome sequencing results of the patient, her parents, and brother. The patient (a) had a heterozygous frameshift mutation in the *PRRT2* gene located in Chr16: 29813703 (NM_145239.2: exon2: c.649dupC (p.Arg217Profs^*∗*^8)); her healthy mother (c) and brother (d) had the same mutation in the *PRRT2* gene; her father (b) had the wild-type *PRRT2* gene.

At 9.7 months of age, the patient underwent a comprehensive examination at the hospital. Laboratory tests and cranial MRI showed no abnormalities. An EEG scan conducted during sleep demonstrated sharp waves in the frontal central area and small spike waves in bilateral central and central midline areas. Whole-exome sequencing was performed on the proband, her parents, and older brother with their informed consent. A mutation in the *PRRT2* gene in the proband was identified (Figure 1(a)), and it was found to have been inherited from the healthy mother (Figure 1(c)). This mutation was also present in her brother (Figure 1(d)), who had also experienced afebrile seizures before the age of two. This mutation was not detected in her father (Figure 1(b)).

**Figure 2:**
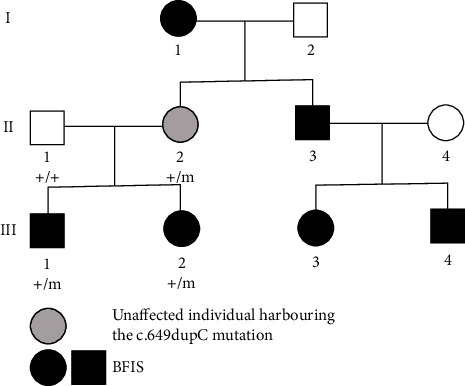
Pedigree of the proband. Patients with the c.649dupC *PRRT2* heterozygous mutation are indicated as +/m, while those tested negative for the mutation are indicated by +/+. BFIS refers to benign familial infantile seizures. III2 is the proband with the *PRRT2* gene mutation and a phenotype of BFIS and autism spectrum disorder. III4 experienced early onset afebrile seizures on the 3rd day of life, received antiepileptic medication before the age of one, went into remission before age 2, and did not undergo whole-exome sequencing testing. I1, II3, and III3 had BFIS before the age of 2, went into remission without medication, and also did not undergo whole-exome sequencing testing.

Considering the classic presentation of seizures, the detection of the same mutation detected in the mother and brother, and the positive family history of other members (Figure 2), a definitive diagnosis of BFIS was made. Recommendation of antiepileptic medication from clinicians was not adopted by her parents. However, at 11.4 months of age, the child experienced convulsions again after a cold, characterized by binocular gaze and slight head shaking, lasting for 10–20 seconds each time, with a frequency of 2 times per day and a duration of 8-9 days. Subsequently, the child was started on levetiracetam therapy (0.14 g, q12h) at 11.8 months of age. However, the seizures did not remit after the initiation of medication. An EEG scan at 15 months of age revealed sharp waves and sharp slow waves in the bilateral frontal pole, frontal area, and frontal midline areas during sleep. Consequently, the dosage of levetiracetam was doubled. Following this adjustment, the convulsions were partially controlled, with occasional episodes lasting about 10 seconds with an asymptomatic interval of 2-3 months.

The child demonstrated normal cognitive and social development up to 15 months of age, achieving milestones such as sitting unaided at 7 months, crawling at 9 months, and uttering four meaningful words (“dad,” “mom,” “grandma,” and “brother”) at 12 months. By 14 months, she was walking independently. However, at 15 months of age, her language and communication skills regressed, and stereotyped and repetitive behaviors emerged, such as pressing buttons and arranging toys, along with difficulties in coping with minor changes. She displayed a poor response to her name, withdrawal, and deteriorating social gaze, with a gradual disappearance of communicative gestures and language. Before the age of two, her parents discontinued her medication due to concerns about potential adverse effects on her neurodevelopment. However, the social, cognitive, and language problems did not show any improvement after the withdrawal of medication. At 2.5 years of age, she could only express four meaningful words (“mom,” “dad,” “open,” and “take”) and exhibited minimal intention to communicate with others. Subsequently, she underwent a comprehensive intellectual and psychobehavioral assessment. Cognitive testing with Gesell Developmental Schedules revealed moderate intellectual disability (ID, DQ = 52.5) and severe receptive and expressive language impairments. The Modified Checklist for Autism in Toddlers was found to be positive for ASD screening. The Childhood Autism Rating Scale supported the diagnosis of ASD. Psycho-Educational Profile-3 also demonstrated severe communication, cognitive, and adaptive behavior impairments. She received rehabilitation for language, social communication, and cognition thereafter. However, abnormal neurodevelopmental delay persisted although seizures remitted since then. At five years of age, she is only able to articulate a few meaningful single words, without forming complete sentences. Throughout this period, her anthropometric measurements have remained within the normal range.

## 3. Discussion

Benign familial infantile seizure (BFIS, OMIM #605751) is an autosomal dominant seizure disorder characterized by a self-limiting course and a benign outcome. Typically, BFIS begins between 3 and 12 months of age and remits spontaneously by 2 years of age [3, 4]. It is often demonstrated as complex generalized or partial tonic-clonic nonfebrile seizures occurring in clusters, with brief spells of motor arrest, cyanosis, limb jerks, eye or head deviation, and generalized hypertonia [4, 5]. Seizures usually had a good response to conventional antiepileptic drugs, with remission rates for treated cases around 98% [1]. BFIS is generally considered to have normal neurodevelopment trajectory and no known long-term neurological sequelae [1, 6]. In some cases, BFIS might be linked to PKD, ICCA, and hemiplegic migraine [1, 6–8].

The main genetic etiology of BFIS has been confirmed in mutations of the *PRRT2* gene on chromosome 16p11.2. Recent data have demonstrated that BFIS, PKD, and ICCA pertain to a common *PRRT2*-associated disease spectrum. Among mutations of the *PRRT2* gene, the frameshift heterozygous mutation (c.649dupC, p.Arg217Profs^*∗*^8) at locus 16p11.2 accounts for approximately 80% of BFIS cases [1, 2, 9, 10]. A deletion at the same position (c.649delC) and at the residue c.291delC accounts for about 4% and 2% of BFIS cases, respectively [1, 10]. Over 95% of cases inherit the mutations from their families, with approximately 1.3% arising from de novo sporadic mutations [1].

The underling mechanism of how *PRRT2* mutations cause BFIS and related disorders remains elusive. Proline-rich transmembrane protein 2 encoded by the *PRRT2* gene is highly expressed in the central nervous system (CNS) and is involved in the modulation of synaptic neurotransmitter release, as well as the synaptic vesicle membrane docking and fusion pathway [7, 11]. Mutations in *PRRT2* produce a truncated protein with a haploinsufficient state, leading to aberrant synaptic neurotransmitter release and neuronal excitability in the CNS [1]. These changes ultimately contribute to the phenotypes of BFIS, with or without paroxysmal movement disorders [1].

However, the genotype-phenotype correlations in *PRRT2*-associated diseases, such as BFIS, have not been fully elucidated. It has been observed that the same mutation in the *PRRT2* gene can result in different phenotypes. For example, in the pedigree of this case, with the same frameshift heterozygous mutation (c.649dupC) in the *PRRT2* gene, the mother was asymptomatic, the elder brother had typical BFIS, and the patient experienced BFIS followed by ASD, ID, and language impairment. In addition, one cousin (III 4 in Figure 2) had an extremely early onset of seizures. These discordant genotype-phenotype presentations of *PRRT2*-associated diseases are hypothesized to result from the combining effects of genetic and environmental factors [1], which need to be elucidated in further studies.

It has been concluded that *PRRT2*-related BFIS typically has a benign prognosis, often resolving with or without medication before the age of 2, and does not result in long-term neurological sequelae and neurodevelopmental delay [1, 6, 7]. While data have shown that both deletions and duplications at the 16p11.2 locus, which encompasses *PRRT2*, are associated with an increased risk for ASD [3, 12], the connection between *PRRT2* mutations and an elevated susceptibility to ASD remains controversial, as deleterious *PRRT2* mutations were not found to be enriched in individuals with ASD [1, 13]. Furthermore, less than 1% of individuals with *PRRT2* heterozygous mutations have ID [1, 4]. However, in this clinically and genetically diagnosed case of BFIS with a *PRRT2* heterozygous mutation, a subsequent follow-up revealed the occurrence of ASD, language impairment, and ID. As far as we know, there have been no reports of ASD regression in cases of BFIS related to heterozygous *PRRT2* mutation (c.649dupC). Roberta Milone et al. reported case of a boy with a deletion in 16p11.2, who initially presented with BFIS and typical neurodevelopment before the age of one. However, he unexpectedly experienced severe autistic regression [3]. In addition, Cossu A et al. described two patients from unrelated families with BFIS carrying the same recurrent *PRRT2* pathogenic variant as our patient in this report. Both patients atypically evolved to encephalopathy related to status epilepticus during sleep (ESES) at preschool age, presenting with developmental regression and behavioral disorders [14]. Furthermore, Diane Vergara et al. reported a case of a 14-year-old girl with a *PRRT2* heterozygous mutation (c.649dupC), who presented with moderate intellectual disability, right hemiparesis, four-limb spasticity, ataxia, dystonia, focal epilepsy and superrefractory status epilepticus (SRSE) following a COVID-19 infection [15].

It is indeed crucial to investigate the underlining mechanism and potential causative factors contributing to the more severe atypical developmental phenotypes observed in our patient and other cases described above. Although reduced penetrance and variable expressivity can to some extent explain the phenotypic variation within families, biallelic *PRRT2* gene variants or concurrent existence of *PRRT2* variants and 16p11.2 microdeletions have been reported in the literature to show more severe phenotypes or have additional clinical features including developmental delay, intellectual disability, and/or autism spectrum disorder [3, 16]. Therefore, a chromosomal microarray analysis should be performed to exclude this possibility. Regrettably, the parents of the child refused the examination due to their residence in another city. Therefore, in relevant cases, concurrent chromosomal microarray analysis and whole-exome sequencing are recommended to comprehensively understand the genotype-phenotype correlation in *PRRT2*-related disorders.

The findings in this report indicate that even recurrent heterozygous mutations, such as the c.649dupC mutation in *PRRT2*, can occasionally give rise to atypical and severe neurodevelopmental outcomes in patients with BFIS [6, 8, 14, 15]. Accordingly, we suggest that infants with BFIS, with or without mutations in *PRRT2*, should be provided with careful clinical follow-up for early detection of possible adverse neurodevelopmental disorders.

## Data Availability

The datasets for this article are not publicly available due to privacy or ethical concerns. Requests to access the datasets should be directed to the corresponding author.
